# Adsorption of Myelin Basic Protein on Model Myelin Membranes Reveals Weakening of van der Waals Interactions in a Lipid Ratio-Dependent Manner

**DOI:** 10.3390/membranes15090279

**Published:** 2025-09-17

**Authors:** Petra Maleš, Barbara Pem, Dražen Petrov, Agustín Mangiarotti, Rumiana Dimova, Danijela Bakarić

**Affiliations:** 1 Division of Organic Chemistry and Biochemistry, Ruđer Bošković Institute, Bijenička 54, 10000 Zagreb, Croatia; petra.males94@gmail.com (P.M.); barbara.pem@irb.hr (B.P.); 2 Institute of Molecular Modeling and Simulation, University of Natural Resources and Life Sciences, 1180 Vienna, Austria; drazen.petrov@boku.ac.at; 3 Max Planck Institute of Colloids and Interfaces, Science Park Golm, 14476 Potsdam, Germany; agustin.mangiarotti@gmail.com (A.M.); rumiana.dimova@mpikg.mpg.de (R.D.)

**Keywords:** model myelin membranes, myelin basic protein, van der Waals interactions, FTIR and CD spectroscopy, differential scanning calorimetry (DSC)

## Abstract

Myelin is a lipid-rich membrane that insulates axons, providing support and ensuring efficient nerve impulse conduction. Disruption of this sheath, or demyelination, impairs neural transmission and underlies symptoms like vision loss and muscle weakness in multiple sclerosis (MS). Despite extensive studies using in vitro and in vivo models, the molecular mechanisms driving demyelination remain incompletely understood. To investigate the role of myelin basic protein (MBP) in membrane stability, we prepared model myelin membranes (MMMs) from lipids expectedly undergoing gel-to-fluid phase transition, mimicking both normal and altered myelin, with and without MBP. Differential scanning calorimetry (DSC) revealed that MBP suppresses the main phase transition in normal MMMs, unlike in modified MMMs. FTIR spectra showed strengthening of van der Waals interactions in normal MMMs with MBP upon heating and opposite effects in the analogous modified MMM system. Additionally, phosphate groups were identified as critical sites for MBP–lipid interactions. Circular dichroism (CD) spectroscopy suggests that MBP adopts helical structures that penetrate the bilayer of normal MMMs. These findings offer new insights into the molecular-level interactions between MBP and myelin membranes, with implications for understanding demyelination in diseases like MS.

## 1. Introduction

Myelin is a lipid-rich extension of the plasma membrane found in oligodendrocytes within the central nervous system (CNS) and Schwann cells in the peripheral nervous system (PNS). It provides structural support and facilitates the efficient transmission of neural impulses. This efficiency is achieved through multiple layers wrapping around the axon, the dense packing of the myelin sheaths, and the very thin layer of water that exists between them [1,2]. Myelin is characterized by high lipid content, constituting 70–85%, compared to proteins, which make up 15–30% [3,4]. Major lipid classes include phospholipids (phosphatidylcholines (PCs), phosphatidylethanolamines (PEs), phosphatidylserines (PSs), and sphingomyelin (SM)), cholesterol (CHOL), and glycosphingolipids such as galactosylcerebroside (GalC) and its sulfatide (GalC-S) form, found in an approximate molar ratio of 40%:40%:20%. The most significant proteins are proteolipid protein (PLP) and myelin basic protein (MBP); the former connects adjacent myelin layers and is a highly conserved hydrophobic protein, whereas the latter is an intrinsically disordered protein (IDP) that acts as an intramembrane adhesive element and makes up 15% of total protein content [3,5,6]. MBP exists in several isoforms, among which 18.5 kDa is the most abundant in the brains of adult humans and cattle [7,8,9], and the enrichment with arginine and lysine residues [10,11] makes it highly positively charged at physiological conditions [12].

A disruption of the spirally wrapped myelin sheath around the axon is referred to as demyelination. This condition encompasses effects such as unwrapping, vacuole formation, and bilayer swelling due to water and ion leakage across the sheaths [13,14,15]. Resulting in the impaired transmission of neural impulses, it produces clinical symptoms such as vision loss and muscle weakness, which are common features of multiple sclerosis (MS) [7,16,17,18]. Studies of experimental autoimmune encephalomyelitis (EAE), an animal model of MS, have demonstrated that the amount and ratios of representative myelin lipids are significantly modified compared to those in normal myelin [19]. In disordered myelin, the lipid composition was found to be significantly perturbed in favor of the increase in PE, PS and CHOL amounts with simultaneous decrease in PC and especially SM [20,21]. The adhesive ability of MBP demonstrated strong dependence on lipid composition [5,22], as well on the presence of certain cations (Ca^2+^) found in a hydrating medium [23].

Despite numerous neuroscience studies devoted to the revelation of the steps that trigger demyelination [24], the efforts that addressed demyelination at the molecular level are, to the best of our knowledge, rather scarce. Gasecka et al. demonstrated the use of Polarized Coherent Raman Microscopy (PR-CARS) to reveal that lipid order changes occur in myelin structures, even when these structures appear morphologically normal at the macroscopic scale [25]. This finding highlights the technique’s ability to uncover molecular-level processes that occur during the early stages of demyelination [25]. A comprehensive study of model myelin membranes (MMMs) by small-angle X-ray scattering (SAXS) and cryo-transmission electron microscopy (cryo-TEM) demonstrated that even minor variations in lipid composition and protein content significantly affect MMM stability and induce structural changes that strikingly resemble those observed in pathological states [26]. PE-enriched MMMs, for example, undergo lamellar (L_α_)-to-inverse hexagonal (H_II_) phase transition at lower temperatures compared to membranes of normal composition, which is further modulated by charged solutes in the aqueous environment [27].

**Figure 1:**
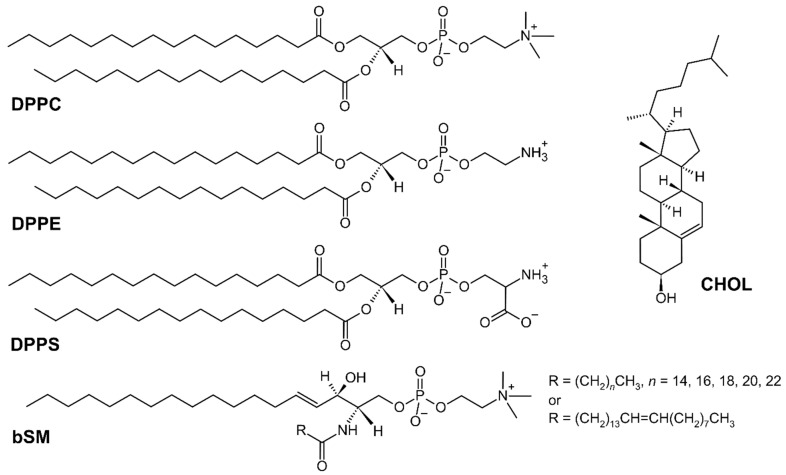
Lipids used for preparation of MMMs: 1,2-dipalmitoyl-*sn*-glycero-3-phosphocholine (DPPC), 1,2-dipalmitoyl-*sn*-glycero-3-phosphoethanolamine (DPPE), 1,2-dipalmitoyl-*sn*-glycero-3-phosphoserine (DPPS), brain sphingomyelin (bSM) and cholesterol (CHOL).

In addition to the previously described influence of negatively charged lipids on structuring or inducing conformational changes in MBP [28,29,30,31,32], Min et al. demonstrated that the altered levels of anionic lipids may deteriorate the adhesive properties of the model myelin membrane [5]. More specifically, the exceptional finding addresses the impact of the amount and ratio of anionic lipids and MBP on the loss of adhesive properties of MMMs, as well as that their swelling is not exclusively reserved for the cytoplasmic leaflet, but that it occurs on the extracellular leaflet as well [5]. To the best of our knowledge, the majority of studies undertaken so far tackle the impact of lipid mono- and bilayer composition on MBP conformation [22], whereas the impact of MBP on the organization of lipids within the bilayers, especially their phase, is scarcely studied. Thus, to address this gap, we investigated the influence of MBP on the thermotropic properties of MMMs, representing normal (healthy) and modified (disordered) myelin, highlighting the considerable increase in PS lipids in the latter [5]. Since the phase of the lipid bilayer made of the zwitterionic lipid 1,2-dipalmitoyl-*sn*-glycero-3-phosphocholine (DPPC) affects the secondary structure of adsorbed MBP [33], we prepared MMMs from lipids that are in the gel phase (L_β(′)_) at room temperature and that can be converted into the fluid phase (L_α_) by heating: DPPC, 1,2-dipalmitoyl-*sn*-glycero-3-phosphoethanolamine (DPPE), 1,2-dipalmitoyl-*sn*-glycero-3-phosphoserine (DPPS), brain sphingomyelin (bSM) and cholesterol (Figure 1). Although the melting temperature (*T*_m_), which is referred to as the peak of L_β(′)_→L_α_ transition (i.e., the main phase transition), is not experimentally accessible for all used lipids (*T*_m_ = 41.5 °C for DPPC, 64.4 °C for DPPE, 51.4 °C for DPPS, 35–40 °C for bSM and 148 °C for CHOL [34,35,36]), using differential scanning calorimetry (DSC) we identified crucial differences in thermotropic properties of model myelin membranes in the presence of MBP, whereas FTIR spectroscopy data provided molecular-level insight into the impact of MBP on van der Waals forces between hydrocarbon chains in MMMs. Ultimately, we explored the dependence of the FTIR response of phosphate groups on the lipid composition and the presence of MBP, probing the hypothesis of strong interactions with positively charged amino acid residues of MBP. Molecular dynamics (MD) simulations were used to test the differences between normal and modified MMMs in terms of the strength of van der Waals interactions, whereas confocal microscopy studies explored the possible aggregation of MMMs in the presence of MBP. The results obtained clearly confirmed that the presence of MBP weakens van der Waals forces between hydrocarbon chains in MMMs in a lipid ratio-dependent manner, and, consequently, that lateral interactions between hydrocarbon chains differ for normal and modified MMMs, as well as in the presence of MBP.

## 2. Experimental

### 2.1. Chemicals and MMM Preparation

DPPC (Avanti, Alabaster, AL, USA, ≥99%), DPPS (Avanti, Alabaster, AL, USA, ≥99%), DPPE (Cayman Chemical, Ann Arbor, MI, USA, ≥98%), bSM (Avanti, Alabaster, AL, USA, ≥99%, porcine), CHOL (Avanti, Alabaster, AL, USA, 98%) and MBP (Sigma-Aldrich, Darmstadt, Germany, 90%, bovine) were purchased as white powders and used as received. Stock solutions of lipids in chloroform (CHCl_3_; colorless liquid, Carlo Erba, Milan, Italy, p.a.) of concentration *γ*(DPPC/DPPS/DPPE/bSM/CHOL) = 10 mg mL^−1^ were prepared and further used in making the films for the preparation of multilamellar liposomes (MLVs) that mimic normal and modified MMMs in the absence and the presence of MBP. Separately, MBP was dissolved in an aqueous solution of NaCl (Kemika, Zagreb, Croatia, p.a. grade; NaCl_(aq)_) of ionic strength *I* = 100 mM (pH ≈ 6) to achieve the mass concentration *γ* = 0.6 mg mL^−1^. After pipetting appropriate volumes of stock solutions of lipids in four flasks (two for normal MMMs and two for modified MMMs), CHCl_3_ was removed from the flasks on a rotary evaporator, and the obtained films were additionally dried under an Ar stream. The films were hydrated with either an aqueous solution of NaCl_(aq)_ or with an aqueous solution of NaCl_(aq)_ containing MBP. The hydration was followed by at least three cycles of suspension vortexing, heating up in a hot H_2_O bath (up to 70 °C), and cooling in an ice bath (4 °C), where each step took 2 min. Ultimately, the mixtures with lipid molar ratios *n*(DPPC):*n*(DDPE):*n*(DPPS):*n*(bSM):*n*(CHOL) = 27:21:4:6:42 for normal MMMs and *n*(DPPC):*n*(DDPE):*n*(DPPS):*n*(bSM):*n*(CHOL) = 19:22:9:2:48 for modified MMMs were obtained. In all four samples, the mass concentration of lipids was *γ* = 5 mg mL^−1^, and the molar ratio of MBP against lipids (in two of four samples) was 1:200 [37]. Lipid suspensions were used as prepared for DSC, FTIR, and confocal microscopy measurements, but were diluted up to *γ* =1 mg mL^−1^ for CD measurements and up to 0.05 mg mL^−1^ for DLS and ELS measurements. The freshly prepared suspensions were examined in all measurements.

### 2.2. Dynamic and Electrophoretic Light Scattering (DLS and ELS) of LUVs: Measurements and Data Analysis

The size distribution of prepared MMMs in the absence and presence of MBP (MMM ± MBP) was determined using dynamic light scattering (DLS) with a photon correlation spectrophotometer (Zetasizer Nano ZS, Malvern Instruments, Worcestershire, UK) that is equipped with a 532 nm green laser. The average hydrodynamic diameter (*d*_H_) was defined as the value at the peak maximum of the volume size distribution. The reported results are the average of six measurements conducted at 25 °C. The zeta (*ζ*) potential was measured by electrophoretic light scattering (ELS) using a Malvern Panalytical Folded Capillary Zeta Cell. This value was calculated from the measured electrophoretic mobility according to the Henry equation, utilizing the Smoluchowski approximation. The measurements were repeated three times for accuracy. Data processing for both DLS and ELS was performed using Zetasizer software version 7.13 (Malvern Instruments) (refer to Supplementary Materials, Figure S1). All suspensions were measured at a concentration of 0.05 mg mL^−1^. Knowing that MBP may exist as a monomer [38], dimer [3] or higher oligomers [31,39], in this research, the term ‘MBP’ designates all possible forms.

The normal and modified MMMs in the absence/presence of MBP were examined with a confocal microscope (Leica TCS-SP8, Leica Microsystems, Mannheim, Germany) equipped with a 63× water immersion objective and with OPSL-488 (Coherent, Berlin, Germany) laser (1%) in a bright field mode at room temperature. All images were stored in LAS X Life Science Microscope Software (Leica Microsystems, Mannheim, Germany, https://www.leica-microsystems.com/products/microscope-software/p/leica-las-x-ls/, accessed on 19 August 2025) and refined in the free Fiji ImageJ software package (v 2) (Figure S2).

### 2.3. Differential Scanning Calorimetry (DSC) of MMMs: Data Acquisition and Curve Analysis

The measurements of normal and modified MMMs in the absence/presence of MBP were conducted in the microcalorimeter Nano-DSC, TA Instruments (New Castle, DE, USA), using the TA Instruments Nano Analyze software (v 3.11.0) package. All suspensions and NaCl_(aq)_ solution were degassed for 10 min before measurements. The suspensions of lipids of normal and modified MMM ± MBP (at lipid concentration of 5 mg mL^−1^) were heated at a scan rate of 1 °C min^−1^ in two repeated heating–cooling cycles in a temperature range of 10–90 °C as duplicates, whereas a reference, NaCl_(aq)_, was heated only once, respectively. Thermotropic properties of normal and modified MMM ± MBP were determined from a thermal history-independent second heating run [40,41] in the following way: the DSC curve of NaCl_(aq)_ was subtracted from the DSC curve of MMM lipid suspensions (both obtained from the second heating run). For further analysis, a temperature range of 30–60 °C was selected, as this encompasses the main phase transition (*T*_m_) of lipid mixtures (DSC curves of individual lipids (DPPC, DPPE, DPPS, bSM and CHOL) are displayed in Figure S3, and DSC curves of MMM ± MBP in the complete temperature range are displayed in Figure S4). After the baseline correction, *T*_m_ was determined from the maximum/maxima of the DSC curve in the examined temperature range.

### 2.4. FTIR Spectra of MMMs: Data Acquisition and Band Analysis

FTIR ATR spectra were measured using an INVENIO-S Bruker spectrometer equipped with a BioATR II unit and a photovoltaic LN-MCT detector, operated with OPUS 8.5 SP1 software (20200710). The KBr beamsplitter was positioned at an aperture of 1 mm, and the detector was set to a scanner velocity of 15 kHz for enhanced sensitivity in measurements. The BioATR II unit was continuously purged with N_2_ gas, and its temperature was maintained using a circulating water bath controlled by a Huber Ministat 125 temperature controller. For the measurements, 20 µL of each sample—MMM (*γ*(MMM) = 5 mg/mL), MBP + MMM (γ(MBP) = 0.6 mg/mL, γ(MMM) = 5 mg/mL), and NaCl_(aq)_—were placed in a small circular sample compartment that utilized dual crystal technology. The FTIR spectra were recorded at temperatures of 30 °C and 60 °C to capture the FTIR spectra of MMMs in (expectedly) predominantly gel (30 °C) and fluid (60 °C) phases. Three independent measurements were conducted for each sample, except for NaCl_(aq)_, which was recorded once. All spectra were acquired with a nominal resolution of 2 cm^−1^ and involved 256 scans.

The acquired spectra were examined in the following spectral ranges: (i) 2980–2820 cm^−1^, which displays the bands originating from the antisymmetric (ν_as_CH_2_) and symmetric (ν_s_CH_2_) stretching of methylene groups in lipid hydrocarbon chains; (ii) 1762–1690 cm^−1^, which contains the band originating from carbonyl stretching of the lipid glycerol backbone (νC=O(OR)); (iii) 1485–1430 cm^−1^, in which the scissoring (γCH_2_) of methylene groups in lipid hydrocarbon chains and the bending of the protonated amino moiety (–NH_3_^+^) of MBP and DPPE appear; and (iv) 1275–1190 cm^−1^, which reveals the band originating from the antisymmetric stretching of phosphate moieties in lipid polar headgroups (ν_as_PO_2_^−^) and of CO groups (ν_as_CO). In the selected ranges, the spectra were smoothed using the Savitzky–Golay procedure (a third-degree polynomial through 30 points), baseline-corrected (using two points at spectral minima), and normalized [42]. Additionally, we examined the region 1800–1480 cm^−1^, which comprises, among other components, the Amide I (C=O(NH)) and Amide II (NH) bands of MBP and bSM, as well as the antisymmetric (ν_as_COO^−^) and symmetric (ν_s_COO^−^) stretching of carboxylic groups of DPPS [43,44]. This spectral range is exceptionally complicated for unambiguous analysis and is displayed in the Supplementary Materials (Figure S5) but omitted from further analysis.

### 2.5. CD Spectra of MBP Adsorbed on MMMs: Measurements and Data Analysis

CD spectra were measured on a Jasco J-815 spectrometer in quartz cells with an optical path of 0.01 cm. 40 µL of MBP (*γ*(MBP) = 1.3 mg mL^−1^, *c*(MBP) = 67.4 μM), MMM (*γ*(MMM) = 1 mg mL^−1^), MBP + MMM (*γ*(MBP) = 0.1 mg mL^−1^; *γ*(MMM) = 1 mg mL^−1^), and NaCl_(aq)_ were put in quartz cells placed in temperature-controlled CD block and measured as at least two separate measurements at temperatures of 30 °C and 60 °C. The CD spectra were recorded in the wavelength range 300–190 nm with the scanning rate of 200 nm min^−1^ and with 2 accumulations. Subtraction of the CD spectrum of MMMs suspended in NaCl_(aq)_ in the absence of MBP from raw CD spectra of MMM + MBP was followed by their smoothing (Savitzky–Golay; polynomial of a 3rd degree and 30 points [42]). The subtracted CD spectra were analyzed using [45].

## 3. Results and Discussion

### 3.1. The Size and Aggregation of MMMs Depend on the Lipid Composition and the Presence of MBP

In the presence of MBP, the average *d*_H_ value of normal MMMs is 3–4 times larger than that of modified MMMs (Figure S1). Additionally, the size range of normal MMM + MBP is significantly broader than that of modified MMM + MBP (see Figure S1). Both phenomena are likely associated with the different interactions between MBP and normal/modified MMMs due to different lipid ratios in the latter, emphasizing the role of anionic PS lipid. Interestingly, in the absence of MBP, normal and modified MMMs are of comparable diameter. *ζ*-potential approaches zero in the presence of positively charged MBP, although this effect is more pronounced in normal MMMs than in modified MMMs owing to the different amount of anionic lipid (Figure S1). Confocal microscopy provided further evidence of protein-driven effects. Imaging showed clear aggregation of MMMs in the presence of MBP, consistent with earlier reports describing MBP’s ability to promote vesicle clustering and membrane adhesion [11,46,47] (Figure S2). These findings support the idea that MBP not only alters the average vesicle size but also drives their aggregation through primarily electrostatic interactions.

### 3.2. MBP Modulates Thermotropic Properties of Normal and Modified MMMs in a Qualitatively Different Manner

In the context of thermotropic properties of lipid membranes, the most distinguished one is the main phase transition or melting, i.e., the transition of lipid bilayers from the gel (L_β(’)_) to fluid (L_α_) phase. The main phase transition has a peak at the melting temperature (*T*_m_), and comprehensive calorimetric studies, subsequently supported by microscopic studies, revealed that the length and (un)saturation degree of hydrocarbon chains of lipid molecules are determining factors in the magnitude of *T*_m_ values [36,48]. More specifically, the main phase transition of lipid bilayers primarily involves reduced van der Waals interactions among hydrocarbon chains, increased hydration of the polar headgroup region, and subsequent thinning of the bilayer [49,50,51,52,53].

**Figure 2:**
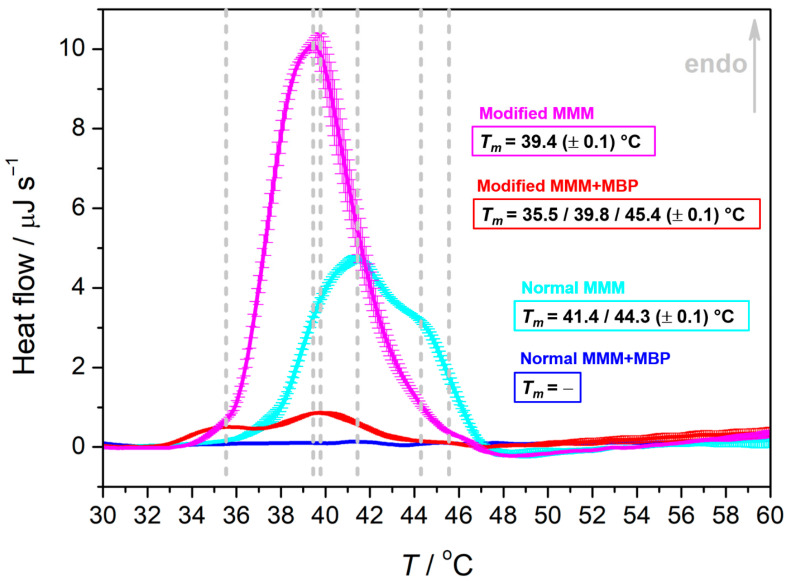
DSC curves of MMM systems in the absence/presence of MBP (blue curve: normal MMM + MBP, cyan curve: normal MMMs; red curve: modified MMM + MBP; magenta curve: modified MMMs) obtained as an average of two measurements with associated uncertainties. Phase transition temperatures are highlighted with dashed lines and are additionally written on graphs and designated with a corresponding color.

The crucial difference between normal MMMs in the presence and absence of MBP is that in the former there are no thermotropic events in the temperature range 30–60 °C, whereas in the latter, two unresolved maxima appear at 41.4 ± 0.1 °C and 44.3 ± 0.1 °C (Figure 2). On the contrary, in modified MMMs in the presence of MBP, DSC curve displays three maxima at 35.5 ± 0.1 °C, 39.8 ± 0.1 °C and 45.4 ± 0.1 °C, while in its absence only one at 39.4 ± 0.1 °C. By comparing these values with those of individual lipids (see Figures S3 and S4), it seems reasonable to assume that the melting of bSM and of DPPC is the dominant process. Moreover, the emergence of multiple transitions implies a more heterogeneous lipid organization in the presence of MBP.

However, what is rather peculiar is that MBP, depending on the lipid composition, may suppress some physicochemical features; in particular, in normal MMMs in the presence MBP, van der Waals forces are reduced even at 30 °C, i.e., when individual lipids are predominantly in the gel phase. This further implies that the interaction of MBP with the lipid membrane surface highly depends on the lipid composition (normal vs. modified) and likely on their distribution. A possible explanation for this is that either MBP in some fashion penetrates the bilayer and interferes with the lipid packing pattern through a direct interaction between particular functional groups or simply that it drastically changes the hydration level of surrounding lipid molecules, which ultimately causes weakening of the van der Waals interactions. These two possibilities are further explored by FTIR and CD spectra of MMM ± MBP systems.

### 3.3. MBP-Induced Weakening of van der Waals Interactions Between Hydrocarbon Chains Depends on Lipid Composition

**Figure 3:**
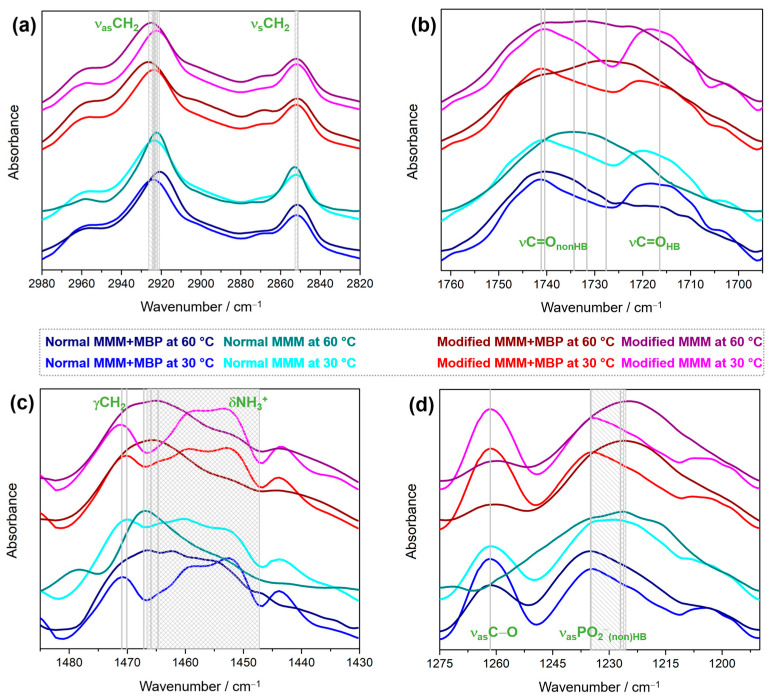
Smoothed, baseline-corrected and normalized (representative) FTIR spectra of normal and modified MMM ± MBP collected at 30 °C and 60 °C in the following spectral regions: (**a**) 2980–2820 cm^−1^ (ν_as_CH_2_ and ν_s_ CH_2_); (**b**) 1765–1690 cm^−1^ (νC=O_(non)HB_); (**c**) 1485–1430 cm^−1^ (γCH_2_ and δNH_3_^+^); (**d**) 1275–1190 cm^−1^ (ν_as_C–O, ν_as_PO_2_^−^_(non)HB_). The spectra were designated as follows: normal MMM + MBP with blue (30 °C) and navy (60 °C), normal MMMs with cyan (30 °C) and dark cyan (60 °C), modified MMM + MBP with red (30 °C) and wine (60 °C), modified MMMs with purple (30 °C) and magenta (60 °C). Note: We would like to draw the reader’s attention to the legend in the middle of the image.

The first examined spectral region (2980–2820 cm^−1^) shows the bands that arise from the (anti)symmetric stretching of CH_2_ groups of hydrocarbon chains (Figure 3a). Regardless of the kind of lipids (in terms of their polar headgroups), the band maxima usually experience a high-frequency shift for about 4 cm^−1^ upon the transformation of the lipids from the gel to fluid phase [54,55,56,57,58], i.e., the weakening of van der Waals interactions [41,59,60]. In this research, an extraordinary phenomenon is detected; for normal MMMs in the presence of MBP the band maximum originated from ν_as_CH_2_ upon heating undergoes a low-frequency shift, i.e., from 2924 cm^−1^ (at 30 °C) to 2921 cm^−1^ (at 60 °C), whereas ν_s_CH_2_ maintains the position of the band maximum at both temperatures (at 2852 cm^−1^ at 30 °C and 60 °C). Contrary to the expected high-frequency shift of both band maxima, this phenomenon implies that upon heating, van der Waals forces between lipid hydrocarbon chains are weaker at 30 °C than at 60 °C. This finding is in line with thermotropic properties (Figure 2) of the normal MMM + MBP system, since the DSC curve does not capture the melting process. Interestingly, in normal MMMs, a similar response is observed; the ν_as_CH_2_ band maximum undergoes a low-frequency shift, i.e., from 2924 cm^−1^ (at 30 °C) to 2922 cm^−1^ (at 60 °C), and ν_s_CH_2_ band maximum remains at the same place at both temperatures (at 2853 cm^−1^ at 30 °C and 60 °C). Although it could be deduced that mere composition of MMMs is the decisive factor for the strength of van der Waals interactions, we would like to draw attention to the DSC curve of normal MMMs. As seen in Figure 2, normal MMMs in the absence of MBP displays the main phase transition, suggesting that the heating weakens van der Waals interaction between hydrocarbon chains, i.e., that they are preserved in the gel phase. Thus, it appears that the factor(s) other than just lipid composition play(s) a role in the appearance of FTIR spectra in this spectral region, which also agrees with the DSC response (more details are elaborated in subsequent subsections). Unlike normal MMM ± MBP, modified MMM ± MBP displays the expected high-frequency shift upon gel-to-fluid phase transition: in the presence of MBP they are displaced of from ν_as_CH_2_ band from 2924 cm^−1^ (at 30 °C) to 2926 cm^−1^ (at 60 °C), while in the absence of MBP from 2922 cm^−1^ (at 30 °C) to 2925 cm^−1^ (at 60 °C), respectively. The maximum of the band originating from ν_s_CH_2_ maintains the position at 2852 cm^−1^ in both gel and fluid phase (at 30 °C and at 60 °C).

The second spectral region (1762–1690 cm^−1^) reveals the bands originating from the stretching of the carbonyl moiety of the glycerol backbone, which can be either free from hydrogen bonding with water molecules from the interfacial layer (νC=O_nonHB_) or involved in a hydrogen bonding (νC=O_HB_) (Figure 3b) [61]. In both normal and modified MMM systems at 30 °C, regardless of the MBP, the spectral envelopes are qualitatively rather similar: in normal MMM + MBP/normal MMMs the maxima assigned as νC=O_nonHB_ and νC=O_HB_ appear at 1741 cm^−1^/1744 cm^−1^ and 1718 cm^−1^/1716 cm^−1^, whereas in modified MMM + MBP/normal MMMs the maxima assigned as νC=O_nonHB_ and νC=O_HB_ appear at 1741 cm^−1^/1740 cm^−1^ and 1721 cm^−1^/1718 cm^−1^, respectively. On the other hand, the spectra at 60 °C display differences attributed to the larger number of maxima in the region in which νC=O_HB_ absorbs (Figure 3b). In particular, in normal MMM + MBP system the maxima assigned as νC=O_nonHB_ are detected at 1741 cm^−1^, and the νC=O_HB_ at 1724 cm^−1^, 1717 cm^−1,^ and 1710 cm^−1^. In normal MMMs, the maximum at 1735 cm^−1^ is a result of a superposition of νC=O_nonHB_ and νC=O_HB_ bands. In modified MMM + MBP, the corresponding maxima appear at 1741 cm^−1^ and 1728 cm^−1^, whereas in modified MMMs the maxima are seen at 1740 cm^−1^, 1734 cm^−1^, 1731 cm^−1^, and at 1723 cm^−1^. The absence of a certain trend in the position and number of band maxima observed in this spectral region suggests that the hydration of the glycerol moiety is a rather delicate function of the amount of bSM [62], as well as on their interaction with CHOL [63,64,65].

As seen from the third spectral region (1485–1430 cm^−1^; Figure 3c), the lateral packing of hydrocarbon chains distinguishes depending on both MMM lipid composition and the presence of MBP: at 30 °C in normal MMM + MBP the band maximum at 1471 cm^−1^ implies that van der Waals forces between hydrocarbon chains are rather weak [55,66], which is opposite for normal MMM system in which the maximum at 1467 cm^−1^ suggest higher order in the packing pattern (more gel-like). The analogous signals in modified MMM + MBP and modified MMMs appear at 1470 cm^−1^ and at 1471 cm^−1^, respectively, suggesting a disorder level comparable to that in normal MMM + MBP. Interestingly, at 60 °C the corresponding band maximum appears at 1467 cm^−1^ (normal MMM + MBP), at 1470 cm^−1^ (normal MMMs), at 1466 cm^−1^ (modified MMM + MBP), and at 1465 cm^−1^ (modified MMMs). The comparison of the values shown by normal MMM + MBP are in line with the data detected in the first examined spectral regions, that van der Waals forces between hydrocarbon chains are weaker at 30 °C than at 60 °C. For normal MMMs, the conclusion drawn from this spectral region is in line with DSC data, which suggests expectedly weaker van der Waals interactions between hydrocarbon chains at 60 °C than at 30 °C [55,66]. The displacement of the wavenumber maxima in the first spectral region of normal MMMs remains, unfortunately, unexplained. Tentatively, the amount of bSM and interdigitation of hydrocarbon chains in normal MMMs may contribute to the observed phenomenon [67,68,69], irrespective to the MBP, but the presence of MBP unambiguously modulates van der Waals interactions between hydrocarbon chains and consequently affects their fluidity at 30 °C and 60 °C. The position of the band maxima at 60 °C in modified MMMs is compromised by the superposition with the bands originating from the bending of –NH_3_^+^ moieties of DPPE and DPPS lipids, as well as of lysine (Lys) residues of MBP [33]. In general, these signals in modified MMM ± MBP systems resemble each other at the corresponding temperature, whereas in normal MMM ± MBP they vary with the presence of MBP and the phase. As previously reported, Lys-originated bands are highly sensitive to the phase of lipid bilayers, while owing to their positive charge, their response is modulated by the amount of anionic lipids [11,16,70,71], among which PS lipids are considerably higher in modified than in normal MMMs [5,70,72].

Besides containing the band originating from the antisymmetric stretching of C–O moiety (ν_as_C–O), the fourth spectral region (1275–1190 cm^−1^) reveals the envelope resulting from the antisymmetric stretching of phosphate groups that are both non-hydrogen-bonded and hydrogen-bonded (ν_as_PO_2_^−^_(non)HB_) (Figure 3d). The former band maximum expectedly appears at 1261–1262 cm^−1^ at 30 °C/1260–1262 cm^−1^ at 60 °C, whereas the latter displays rather different temperature/phase-dependent features depending on the MMM composition and the presence of MBP. In normal MMM ± MBP systems, the position of the envelope maximum barely displaces upon the phase change, remaining at 1235 cm^−1^ after heating, while in normal MMMs it exhibits a small low-frequency shift (from 1228 cm^−1^ to 1226 cm^−1^). A change in the envelope appearance at the low-frequency side suggests that the hydration of phosphate groups of lipid molecules at 60 °C in normal MMMs is not only different from that in normal MMM + MBP, but also more heterogeneous (several maxima are seen). At this stage, it seems reasonable to conclude that the adsorption of MBP onto normal MMMs and the interaction of positively charged residues with phosphate groups attenuate the hydration of phosphate groups. On the contrary, in modified MMM ± MBP systems, the envelopes qualitatively resemble each other and also exhibit a considerable phase transition-induced low-frequency shift in the envelope maximum; in modified MMM + MBP, the maximum displaces from 1234 cm^−1^ (30 °C) to 1226 cm^−1^ (60 °C), whereas in modified MMMs from 1234 cm^−1^ (30 °C) to 1225 cm^−1^ (60 °C). Overall, it seems straightforward to conclude that the hydration of phosphate groups in normal and modified MMMs is a function of the total lipid charge and distribution, and that the presence of MBP, depending on the composition of MMMs (normal vs. modified), may additionally perturb the hydration pattern by engaging electrostatically with headgroups.

Owing to the obscurity of Amide I/II bands by the signals emerged from bSM (and CHOL) [73] (see Figures S5 and S6), the unambiguous analysis from linear FTIR spectra [74,75] of was prevented so they are not included in the data analysis and interpretation.

### 3.4. Secondary Structure of MBP Depends on MMM Composition and the Phase

**Figure 4:**
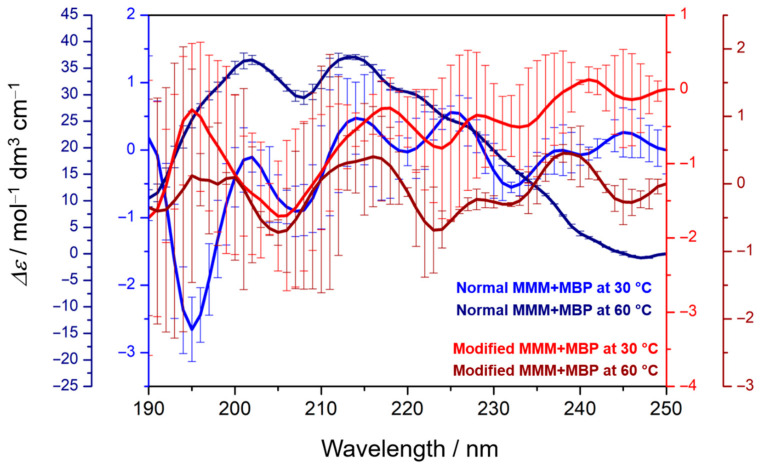
CD spectra of: normal MMM + MBP at 30 °C/60 °C (blue/navy); modified MMM + MBP at 30 °C/60 °C (red/wine).

Following the CD spectra of MBP adsorbed on normal and modified MMMs (Figure 4), it is clear that only the normal MMM at 30 °C presents the expected CD spectrum of an intrinsically disordered protein such as MBP (blue curve with maximum at about 190 nm). Upon heating, the spectrum becomes positive, suggesting the presence of helical structures (navy curve). In modified MMMs, regardless of the global phase of lipids, the spectra resemble one another (red and wine curves). The maxima/minima at about 208 nm and 220 nm suggest that MBP in the modified MMM system is not as disordered as in a normal MMM system, and there is a certain amount of alpha-helical structures [32,76]. Considering that the BestSel [45] reported the presence of helical structures for normal MMM + MBP at 60 °C only (Figure S7), despite of large uncertainties, it seems more likely that MBP forms a differently disordered structure when adsorbed onto normal MMMs than onto modified MMMs. When elaborating the structuring of MBP, an important issue that emerges from the conducted experiments is the temperature range in which the systems were prepared, i.e., the expected gel-to-fluid phase transition of the used lipids requires temperatures higher than physiological ones. As the heating makes IDPs transformation from one distribution of secondary structures to another one [77,78], we expect that temperatures as high at 60 °C will not decompose MBP. Moreover, in comparison with CD spectra of MBP dissolved in aqueous medium, where it displays only the signal at 195 nm (see Figure S8, data taken from [33]), MBP adsorbed onto MMMs only changes the distribution of secondary structures, which, according to Figure 4 (and Figure S7), depends on the MMM composition. In turn, this means that the lipid composition is a primary, and the lipid bilayer phase is a secondary factor in dictating MBP secondary structure [11,16,31,33,71].

### 3.5. Implications of MBP Adsorption on the Changes in Lipid Membrane Fluidity and Aggregation

Since the adsorption of MBP on normal MMMs clearly weakens van der Waals interactions between hydrocarbon chains at 30 °C and then slightly increases them at 60 °C (but maintaining the fluid phase), the finding emerging from this phenomenon directly tackles the variability in the bilayer fluidity. Other researchers have already recognized the importance of this issue. For instance, when exploring myelin-like monolayers that mimic the cytoplasmic leaflet that exclusively contains PS lipids, Widder et al. found that electrostatic interactions between negatively charged PS-containing monolayers and MBP lowered the fluidity of myelin-like monolayers [20], but also that the amount of bSM may be critical for maintaining fluidity [16]. More specifically, bSM introduces complexity to the organization of lipid bilayer phases due to the asymmetry of the hydrocarbon chains (Figure 1) and the accompanying interdigitation [79]. Using structural and microscopic techniques, Krugmann et al. revealed that the interaction of MBP with lipids in myelin varies with the composition, which may increase their instability and enhance local curvature [80]. In particular, they found that the diseased myelin has lower bending rigidity and experiences high local curvature [81], which was also potentially associated with variable bSM content [80].

Nevertheless, when CD data are compared along with DSC and FTIR results, especially for normal MMMs in the presence of MBP at 60 °C, it suggests that a certain fraction of helical structures of MBP may be formed upon interactions with lipid membranes. A certain part of MBP may penetrate the bilayers, causing overall stiffening of the normal MMM (lower band maximum at 60 °C than at 30 °C) [30]. At both temperatures, the van der Waals interactions are clearly weaker in normal than in modified MMMs, but MBP potentially inserts into the membrane, making overall packing more compact than mere normal MMMs.

MD simulations of the normal and modified systems were, unfortunately, only feasible in the absence of MBP. MBP, as an intrinsically disordered protein, does not have a well-defined initial conformation, and due to the known sampling limitations of MD (difficulties in achieving convergence), any membrane interaction will be heavily biased by the initial setup. Since adequate exploration of MBP conformational space would require separate enhanced sampling simulations and the usage of different force fields, it was declared to be out of the scope of this study [82,83,84]. The simulations of MMMs alone, especially the results addressed to the maintenance of van der Waals interactions at 30 °C and 60 °C) are presented in Supplementary Materials (Section S5).

In the context of the impact of MBP on lipid-dependent MMM fluidity, the results provided from the reported studies may be in line with our observation as well. However, some important differences should be highlighted. The MBP-induced aggregation appears to be a critical factor in the development not only of MS [38], but also of other pathological states [85,86,87]. In particular, the aggregation of lipid bilayers in the presence of MBP is expected to be the function of lipid ratio [11], but our microscopic experiments were not able to elucidate the differences between the aggregation of normal and modified MMMs in the presence of MBP. A potential cause for the lack of information in this context may arise from the size of lipid bilayers since we worked with MLVs as MMMs, whereas Mac Millan et al. used large unilamellar vesicles (LUVs) [11]. Finally, besides the fact that some authors highlight the importance of certain lipids like bSM for mono- or bilayer fluidity [38], it seems reasonable to assume that the altered amount of CHOL also contributes to the modulation of van der Waals forces and overall bilayer fluidity [88,89]. Owing to the complexity of this research, these results will be presented in a subsequent study.

## 4. Conclusions

By building up the MMMs from the same lipids, but at different ratios, we revealed that van der Waals forces between hydrocarbon chains are considerably weaker for the lipid composition that mimics normal myelin as opposed to that of modified myelin. DSC study confirmed the absence of any thermotropic event in normal MMMs in the presence of MBP, whereas FTIR spectra suggest that for normal MMM ± MBP van der Waals interactions are substantially weak at both 30 °C and 60 °C (from the bands originating from antisymmetric stretching of methylene groups). However, for normal MMMs in the presence of MBP, the additional confirmation of van der Waals forces weakness at both temperatures emerges from the signal originating from scissoring of methylene groups that indicate no change in the lateral arrangement between lipid molecules at both temperatures. Moreover, the hydration of phosphate groups of lipids in normal MMMs is substantially different from that in modified MMMs. In the context of CD measurements, it is suggested that MBP has a different disordered structure when adsorbed at the surface of normal and modified MMMs. Owing to the possibility of displaying a certain amount of helical structure in normal MMMs at 60 °C, its hydrophobic parts may insert into the bilayer and stiffen it, which coincides with heating-induced displacement of the maximum of antisymmetric stretching of methylene groups. To the best of our knowledge, this manuscript is the first example that highlights the molecular-level insight into the lipid ratio-dependent weakening of van der Waals interactions between hydrocarbon chains that directly impacts the fluidity and tackles aggregation-induced effects that emerge upon the adsorption of IDP.

## Data Availability

The original contributions presented in this study are included in the article/Supplementary Material. Further inquiries can be directed to the corresponding author.

## Supplementary Materials

The following supporting information can be downloaded at: https://www.mdpi.com/article/10.3390/membranes15090279/s1, Figure S1: The size and *ζ*-potential data of normal and modified MMM and the absence and presence of MBP obtained at 25 °C; Figure S2: Confocal microscope images of MMM in bright field (scale bars: 2 and 20 µm as indicated on the images): (a) normal MMM + MBP; (b) modified MMM + MBP; (c) normal MMM; (d) modified MMM; Figure S3. DSC curves of individual lipids with highlighted phase transition(s) temperature(s) (DPPC-red; DPPS-blue; DPPE-green, bSM-cyan; CHOL-gray); Figure S4. DSC curves of normal and modified MMM in the absence and presence of MBP (normal and modified MMM ± MBP) in the 10–90 °C temperature range; Figure S5. FTIR spectra of normal and modified MMM in the absence and presence of MBP (normal and modified MMM ± MBP) in the spectral range 1800–1480 cm^−1^ acquired at 30 °C and 60 °C (the legend is displayed on the right side of the figure); Figure S6. FTIR spectra of bSM (a) and MBP (b) in the spectral range 1800–1480 cm^−1^ acquired at 20 °C and 50 °C; Figure S7. Fractions of secondary structures in normal and modified MMM in the absence and presence of MBP (normal and modified MMM ± MBP) obtained from BestSel; Figure S8. Experimental CD spectra of MBP dissolved in NaCl_(aq)_ at 20 °C and 50 °C; Figure S9. Deuterium order parameters (-S_CD_) of palmitic acid chains within lipids in normal and modified MMM, at 30 °C and 60 °C; Figure S10. Final snapshots of the simulated systems, with a representative lipid of each type (DPPC—blue, DPPE—orange, DPPS—green, SSM—purple, CHOL—black) highlighted: top left—normal MMM at 30 °C, top right—modified MMM at 30 °C, bottom left—normal MMM at 60 °C, bottom right—modified MMM at 60 °C; Table S1: Structural parameters of model membranes: area per lipid (APL), membrane thickness (MT) and lateral diffusion coefficient (D).
