# Supplementary material for: Adsorption of Myelin Basic Protein on Model Myelin Membranes Reveals Weakening of van der Waals Interactions in a Lipid Ratio-Dependent Manner
Source: Membranes (Basel). 2025 Sep 17;15(9):279. doi: 10.3390/membranes15090279 (PMC12471910; doi:10.3390/membranes15090279)
Supplement: Supplementary file 1 [file membranes-15-00279-s001.zip › membranes-3843383-supplementary.pdf]

Adsorption of myelin basic protein on model myelin membranes reveals weakening of van der Waals interactions in a lipid ratio-dependent manner

Petra Maleš[a], Barbara Pem[a], Dražen Petrov[b], Agustín Mangiarotti[c], Rumiana Dimova[c], Danijela Bakarić[a, *]

[a] Division of Organic Chemistry and Biochemistry, Ruđer Bošković Institute, Bijenička 54, 10000 Zagreb, Croatia

[b] Institute of Molecular Modeling and Simulation, University of Natural Resources and Life Sciences, 1180 Vienna, Austria

[c] Max Planck Institute of Colloids and Interfaces, Science Park Golm, 14476 Potsdam, Germany

*Corresponding author. Tel.: +385 1 4571 382; E-mail: danijela.bakaric@irb.hr (Danijela Bakarić)

Supporting Information

S1. DLS, ζ-potential and microscopic data on MMM p2

S2. Additional DSC data on individual lipids and MMM p3

S3. Additional FTIR data on MMM, bSM and MBP p4

S4. Additional CD data on MMM and MBP p5

S5. Molecular dynamics data p6

References p9

S1. DLS, ζ-potential and microscopic data on MMM

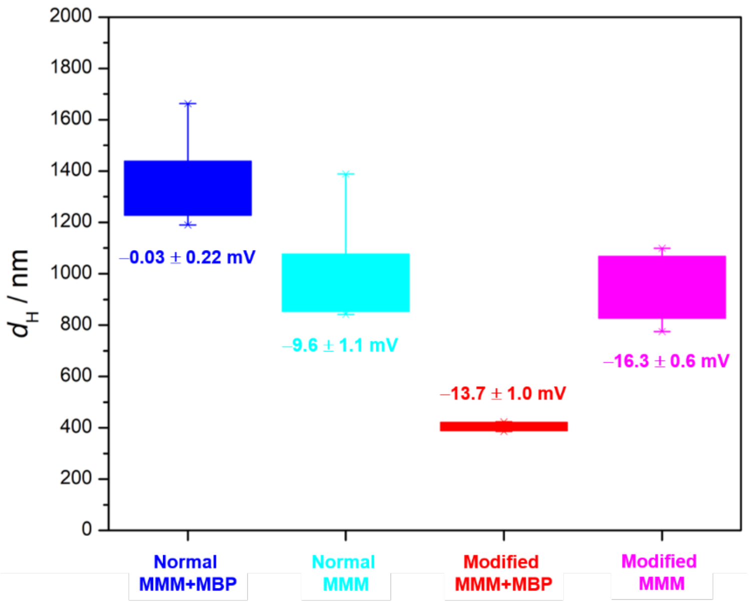

Figure S1. The size and ζ-potential data of normal and modified MMM and the absence and presence of MBP obtained at 25 °C.

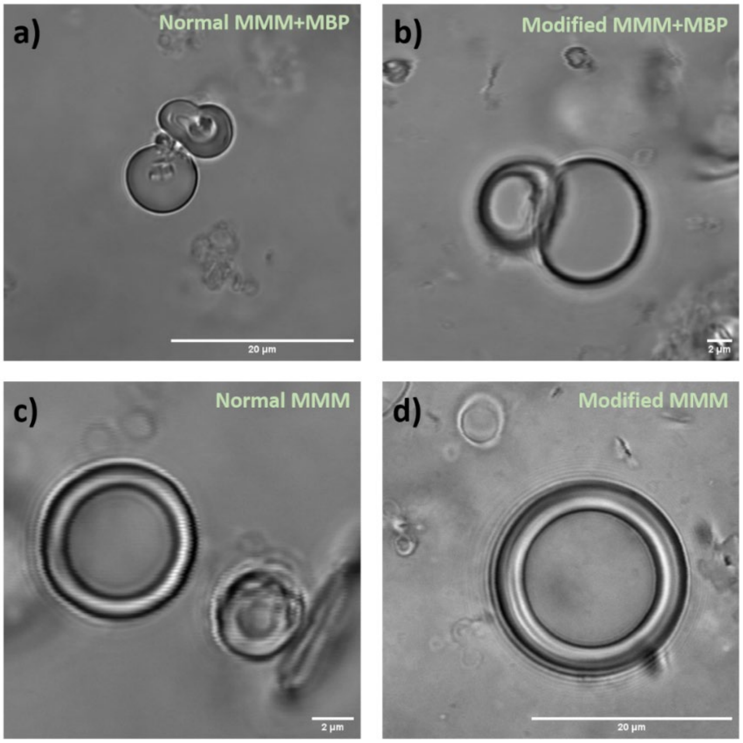

Figure S2. Confocal microscope images of MMM in bright field (scale bars: 2 and 20 μm as indicated on the images): a) normal MMM + MBP; b) modified MMM + MBP; c) normal MMM; d) modified MMM.

S2. Additional DSC data on individual lipids and MMM

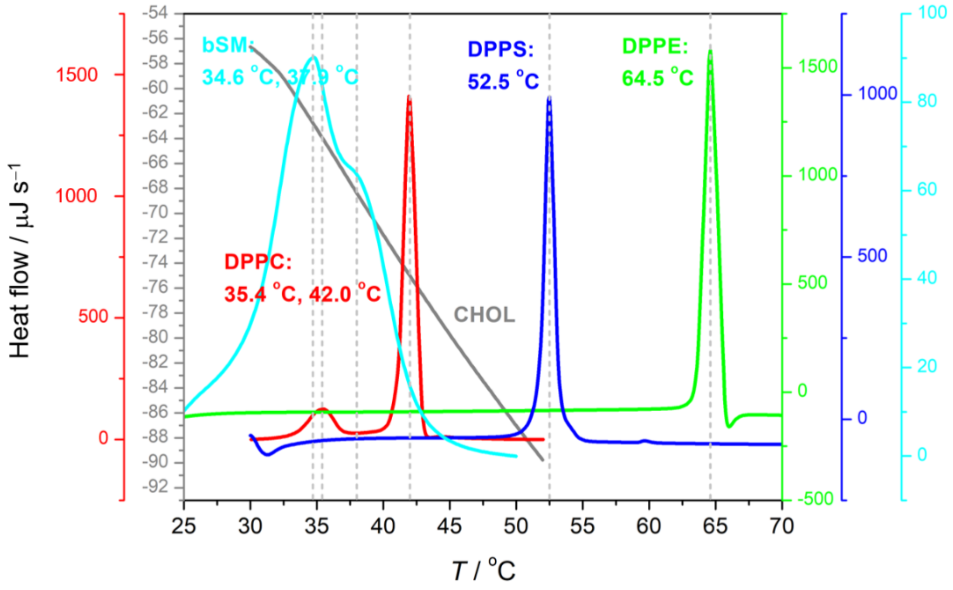

Figure S3. DSC curves of individual lipids with highlighted phase transition(s) temperature(s) (DPPC-red; DPPS-blue; DPPE-green, bSM-cyan; CHOL-gray) (data taken from [1–4]).

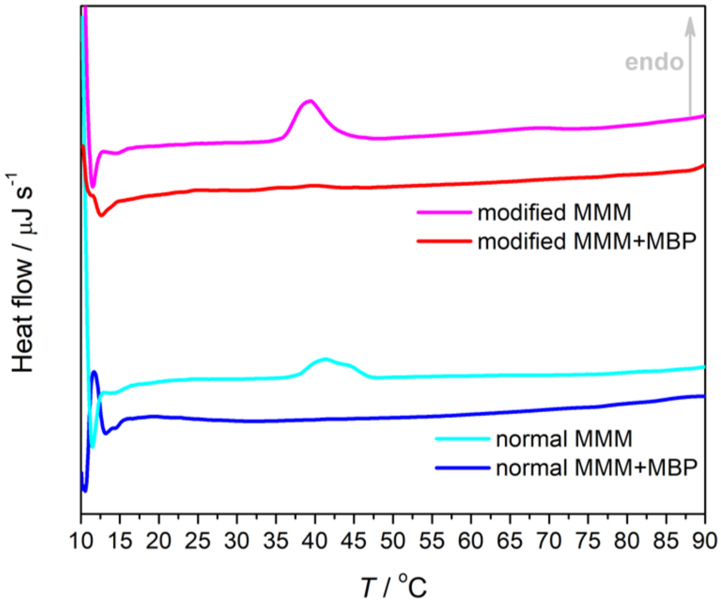

Figure S4. DSC curves of normal and modified MMM in the absence and presence of MBP (normal and modified MMM ± MBP) in the 10-90 °C temperature range.

S3. Additional FTIR data on MMM, bSM and MBP

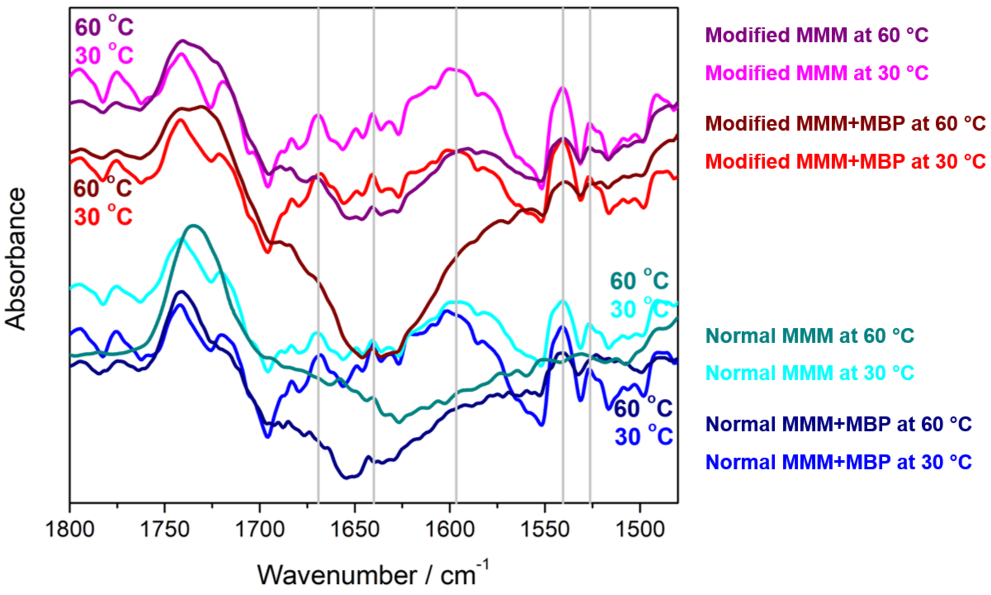

Figure S5. FTIR spectra of normal and modified MMM in the absence and presence of MBP (normal and modified MMM ± MBP) in the spectral range 1800-1480 cm^{-1} acquired at 30°C and 60 °C (the legend is displayed on the right side of the figure).

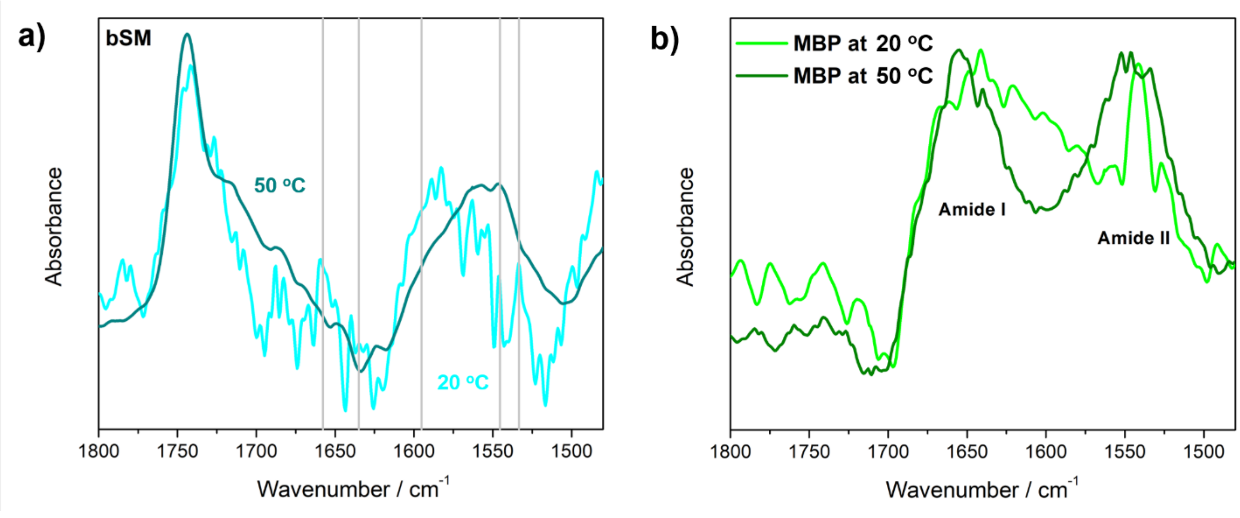

Figure S6. FTIR spectra of bSM (a) and MBP (b) in the spectral range 1800-1480 cm^{-1} acquired at 20°C and 50 °C (data taken from [4] (a) and [5] (b)).

S5. Additional CD data on MMM and MBP

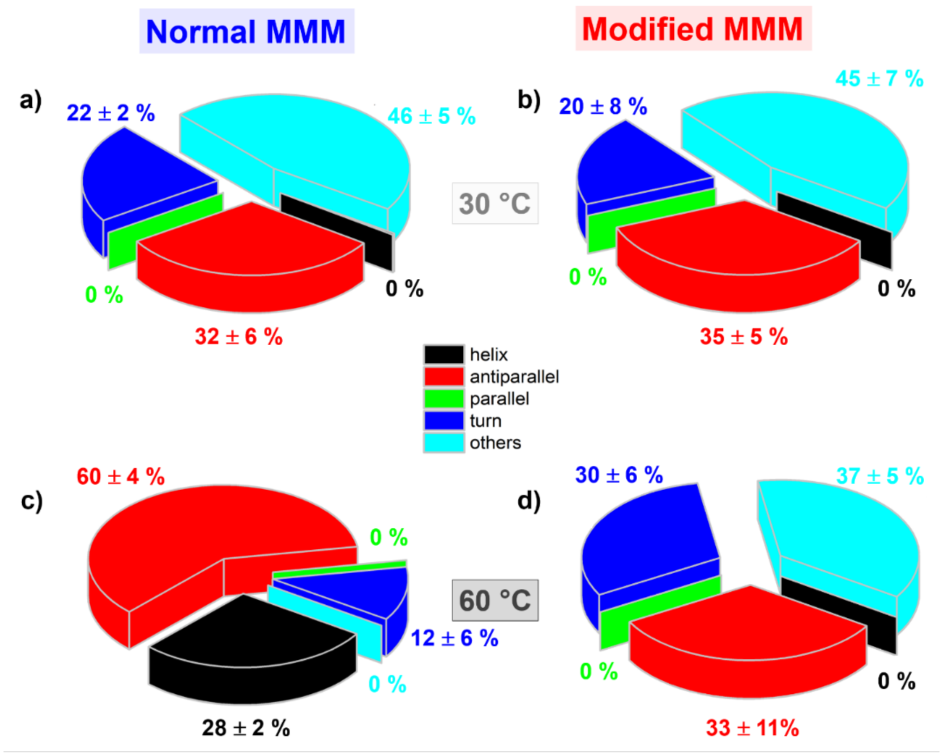

Figure S7. Fractions of secondary structures in normal and modified MMM in the absence and presence of MBP (normal and modified MMM ± MBP) obtained from BestSel [6].

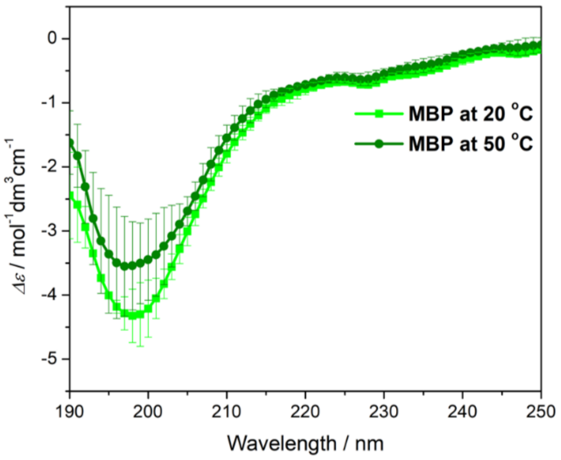

Figure S8. Experimental CD spectra of MBP dissolved in $NaCl_{(aq)}$ at 20 °C and 50 °C (data taken from [5]).

S6. Molecular dynamics data

Methods

Classical molecular dynamics (MD) was employed to study MMMs in gel or fluid phases. The model membranes consisted of 400 lipid molecules in the following ratios: 104 DPPC, 116 DPPE, 28 DPPS, 24 stearoyl-SM (SSM) and 128 CHOL molecules for the normal MMM; 80 DPPC, 132 DPPE, 28 DPPS, 8 SSM and 152 CHOL molecules for the modified MMM. The membranes were solvated with 35 waters per lipid, and 52 Na^+ and 24 Cl^- were added to neutralize the system and achieve experimental ionic strength. The membranes were prepared using the CHARMM-GUI Membrane Builder module [7], and the simulations were run in GROMACS 2020.0 [8]. CHARMM36m force field [9] was used to describe the lipids, as well as the TIP3P model [10] for water. The bilayers were equilibrated at 30 °C and 60 °C following the standard CHARMM-GUI procedure, and underwent a 500 ns production run in the NpT ensemble. The temperature was controlled with the Nosé-Hoover thermostat (time constant of 1 ps), and the pressure with the Parrinello-Rahman barostat (semi-isotropic pressure coupling, time constant of 5 ps, target pressure 1 bar). Particle mesh Ewald (PME) was used for long-range Coulomb interactions. The short-range interactions cutoff was 1.2 nm with a switching function activated at 1.0 nm. Bonds with hydrogen were constrained with LINCS. 3D periodic boundary conditions were used throughout. The time step was 2 fs. Data analysis and visualization were conducted using GROMACS modules, APL@Voro [11] and Visual Molecular Dynamics (VMD) [12]. The equilibration period of the production runs was determined based on the stabilization of the membrane surface (defined by x and y box lengths), and the first 200 ns were omitted from the analysis. All results are an average of 3 independent replicates.

Results

Classical MD was employed to evaluate the difference in structural properties between MMM at 30 °C and 60 °C, as it can provide insight into molecular-level events and organization. Simulating the membranes below and above the transition temperature serves to confirm the changes in lipid organization that are indicative of modified van der Waals interactions. Basic structural parameters of the lipid membranes are given in Table S1.

Table S1. Structural parameters of model membranes: area per lipid (APL), membrane thickness (MT) and lateral diffusion coefficient (D).

System		APL / nm^2	MT / nm	D / $cm^2\,s^{-1}$
Normal MMM	30 °C	0.393 ± 0.001	4.581 ± 0.011	$2.6*10^{-9} \pm 2.3*10^{-10}$
	60 °C	0.425 ± 0.002	4.431 ± 0.016	$5.6*10^{-8} \pm 1.8*10^{-8}$
Modified MMM	30 °C	0.393 ± 0.001	4.466 ± 0.010	$7.8*10^{-9} \pm 3.3*10^{-9}$
	60 °C	0.420 ± 0.002	4.348 ± 0.014	$4.4*10^{-8} \pm 1.4*10^{-8}$

Area per lipid (APL) was determined by dividing the *xy* box surface with the number of lipids in one leaflet. It is the main indicator of lipid packing, and the lower values at 30 °C and higher values at 60 °C are reflective of the packing differences in gel and fluid phases. Interestingly, there is no difference in APL in the gel phase between normal and modified MMM, despite the difference in composition. Membrane thickness (MT) was calculated as the distance between P atoms in opposing leaflets. It also shows the difference in lipid phase, since the less structured fluid membrane features lipid tail interdigitation, which reduces MT. Here, the difference between normal and modified MMM is visible – the latter are thinner, likely because they contain more CHOL, and less lipids with longer acyl chains. The other indicator of membrane fluidity is the lateral diffusion coefficient (D), which is a measure of lipid mobility within the membrane. Naturally, it is larger in fluid membranes (here by an order of magnitude). It was obtained from the slope of the mean square displacement in dependence of time. Finally, deuterium order parameters ($-S_{CD}$, Figure S9), calculated for the chains of palmitic acid, which is most abundant in the observed system, show the difference in acyl chain orientation, with lower values indicating a higher level of disorder. In the gel phase, lipid chains are tightly packed and mostly straight due to the strong van der Waals interactions between them, while in the fluid phase, they are more disordered with larger conformational freedom due to the weakening of those forces.

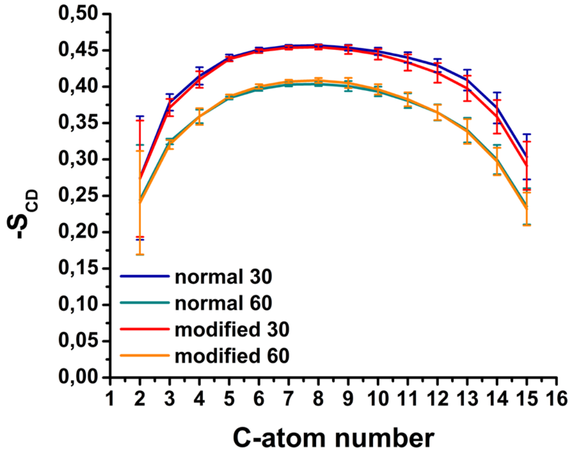

Figure S9. Deuterium order parameters ($-S_{CD}$) of palmitic acid chains within lipids in normal and modified MMM, at 30 °C and 60 °C.

Ultimately, the MD simulations have confirmed the phase difference between MMM at simulation temperatures and provided confirmations of van der Waals forces weakening after phase transition. The differences in the membranes in gel and fluid phases are further visualized in Figure S10.

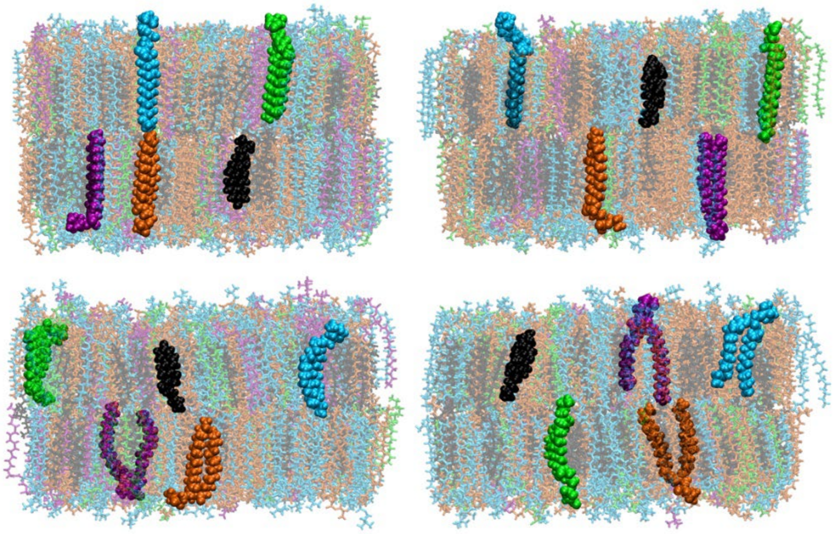

Figure S10. Final snapshots of the simulated systems, with a representative lipid of each type (DPPC – blue, DPPE – orange, DPPS – green, SSM – purple, CHOL – black) highlighted: top left – normal MMM at 30 °C, top right – modified MMM at 30 °C, bottom left – normal MMM at 60 °C, bottom right – modified MMM at 60 °C.

References

1. Maleš, P.; Brkljača, Z.; Domazet Jurašin, D.; Bakarić, D. New Spirit of an Old Technique: Characterization of Lipid Phase Transitions via UV/Vis Spectroscopy. *Spectrochim. Acta Part A Mol. Biomol. Spectrosc.* **2022**, *272*, 121013, 7, doi:10.1016/j.saa.2022.121013.

2. Maleš, P.; Butumović, M.; Erceg, I.; Brkljača, Z.; Bakarić, D. Influence of DPPE Surface Undulations on Melting Temperature Determination: UV/Vis Spectroscopic and MD Study. *Biochim. Biophyica Acta Biomembr.* **2023**, *1865*, 184072, 9.

3. Pašalić, L.; Pem, B.; Bakarić, D. Lamellarity-Driven Differences in Surface Structural Features of DPPS Lipids: Spectroscopic, Calorimetric and Computational Study. *Membranes (Basel).* **2023**, *13*, 83, 22.

4. Maleš, P.; Munivrana, J.; Pašalić, L.; Pem, B.; Bakarić, D. Reorientation of Interfacial Water Molecules during Melting of Brain Sphingomyelin Is Associated with the Phase Transition of Its C24:1 Sphingomyelin Lipids. *Chem. Phys. Lipids* **2024**, *264*, 105434, 10, doi:10.1016/j.chemphyslip.2024.105434.

5. Maleš, P.; Brkljača, Z.; Crnolatac, I.; Petrov, D.; Bakarić, D. Phase-Dependent Adsorption of Myelin Basic Protein to Phosphatidylcholine Lipid Bilayers. *Membranes (Basel).* **2024**, *14*, 15, 19, doi:10.3390/membranes14010015.

6. BeStSel^{TM} (2014-2023) – ELTE Eötvös Loránd University, Budapest, H. BeStSel. https://bestsel.elte.hu/index.php (08.05.2025).

7. Wu, E.L.; Cheng, X.; Jo, S.; Rui, H.; Song, K.C.; Dávila-Contreras, E.M.; Qi, Y.; Lee, J.; Monje-Galvan, V.; Venable, R.M.; et al. CHARMM-GUI Membrane Builder Toward Realistic Biological Membrane Simulations. *J. Comput. Chem.* **2015**, *35*, 1997–2004, doi:10.1002/jcc.23702.CHARMM-GUI.

8. Abraham, M.J.; Murtola, T.; Schulz, R.; Páll, S.; Smith, J.C.; Hess, B.; Lindah, E. Gromacs: High Performance Molecular Simulations through Multi-Level Parallelism from Laptops to Supercomputers. *SoftwareX* **2015**, *1–2*, 19–25, doi:10.1016/j.softx.2015.06.001.

9. Klauda, J.B.; Venable, R.M.; Freites, J.A.; O'Connor, J.W.; Tobias, D.J.; Mondragon-

Ramirez, C.; Vorobyov, I.; MacKerell Jr, A.D.; Pastor, R.W. Update of the CHARMM All-Atom Additive Force Field for Lipids: Validation on Six Lipid Types. *J Phys Chem B* **2010**, *114*, 7830–7843, doi:10.1021/jp101759q.Update.

10. Jorgensen, W.L.; Chandrasekhar, J.; Madura, J.D.; Impey, R.W.; Klein, M.L. Comparison of Simple Potential Functions for Simulating Liquid Water. *J. Chem. Phys.* **1983**, *79*, 926–935.

11. Kern, M.; Jaeger-Honz, S.; Schreiber, F.; Somme, B. APL@voro—Interactive Visualization and Analysis of Cell Membrane Simulations. *Bioinformatics* **2023**, *39*, btad083, 3.

12. Humphrey, W.; Dalke, A.; Schulten, K. VMD: Visual Molecular Dynamics. *J. Mol. Graph.* **1996**, *14*, 33–38, doi:10.1016/j.carbon.2017.07.012.
